# Special Issue ‘’Pre-Clinical Studies of Personalized Medicine for Cancer Research’’

**DOI:** 10.3390/cancers17162607

**Published:** 2025-08-08

**Authors:** Nuno Vale

**Affiliations:** 1 PerMed Research Group, RISE-Health, Faculty of Medicine, University of Porto, Alameda Professor Hernâni Monteiro, 4200-319 Porto, Portugal; nunovale@med.up.pt; 2 Laboratory of Personalized Medicine, Department of Community Medicine, Health Information and Decision (MEDCIDS), Faculty of Medicine, University of Porto, Rua Doutor Plácido da Costa, 4200-450 Porto, Portugal; 3 RISE-Health, Department of Community Medicine, Health Information and Decision (MEDCIDS), Faculty of Medicine, University of Porto, Rua Doutor Plácido da Costa, 4200-450 Porto, Portugal

The concept of personalized medicine (PM) has emerged as a prominent area of research to complement conventional medical approaches, grounded in the understanding that the underlying heterogeneity of individuals at the molecular, physiological, environmental exposure, and behavioral levels must be addressed by implementing interventions that are tailored or ‘personalized’ to these unique and nuanced characteristics. Clinical outcomes from traditional methods have shown that the ‘one size fits all’ approach should be replaced. PM in cancer research considers both inter- and intra-tumor variability in genes, tumor environment, and lifestyle, as well as the comorbidities of each person diagnosed with cancer. The ultimate goals are to optimize tumor response, reduce therapy-induced toxicity, and ensure better patient quality of life and well-being. Pre-clinical studies emerge within this same rationale: to provide valuable insights into the efficacy, safety, and potential adverse effects of personalized interventions before they are tested in clinical trials. In this context, the present Special Issue of *Cancers* aims to discuss and present the latest pre-clinical studies in personalized medicine for cancer research.

Cancer genomics is a fundamental component of personalized medicine in oncology, enabling the identification of genetic mutations that drive tumor development, particularly with the widespread adoption of next-generation sequencing (NGS). Mechahougui et al. [1] explored the direct associations between genomic alterations and targeted therapies, examining the key genetic factors, challenges in clinical application and research, and strategies to enhance the therapeutic efficacy of precision oncology. The landscape of cancer therapy is rapidly evolving, shifting from traditional organ-based classifications to a genomics-driven approach that transcends tumor origin. This shift is exemplified by recent breakthroughs, such as the development of KRAS G12C inhibitors, which target mutations once considered ‘undruggable’, and the growing potential to therapeutically address TP53 mutations. NGS has played a pivotal role in enabling these advances by facilitating the identification of actionable mutations and supporting a transition toward gene-directed therapies. Reflecting this transformation, clinical trial designs are increasingly organized around molecular profiles rather than tumor histology, as demonstrated by innovative studies mentioned by the authors. In addition, NGS testing is also being employed in early-phase clinical trials. For this, samples need to meet quantitative and qualitative standards. Esposito et al. [2] retrospectively examined the records of patients referred to the Early Drug Development (EDD) Unit of the European Institute of Oncology who underwent biopsies for research purposes to assess the safety of biopsy procedures and the adequacy of the samples for NGS testing. Among 731 patients, nearly half underwent mandatory biopsies for research, with a diagnostic yield of 98% and an NGS success rate of 88.4%. Despite the overall efficacy, a notable proportion of NGS failures stemmed from poor sample quality or insufficient material. The non-negligible failure rate of NGS testing underscores the need for implementing specific guidelines and Standard Operating Procedures for samples intended for NGS. Further highlighting the clinical utility of NGS, Ramos et al. [3] emphasize its critical role in advancing personalized medicine in lung cancer, particularly in non-small cell lung cancer (NSCLC). These authors explore the current therapeutic approaches for various lung cancer types and underscore recent progress in emerging targeted therapies. These developments support the adoption of precision oncology in clinical practice by aligning treatment decisions with specific molecular alterations, ultimately improving patient outcomes. Still within the scope of lung cancer, tumors of the pleura, such as metastatic lung cancer and mesothelioma, remain among the most lethal and therapy-resistant malignancies. Ötvös et al. [4] isolated and cultured cells from pleural effusions in three-dimensional (3D) cell aggregates and compared their ex vivo drug sensitivity to the clinical response to the same chemotherapeutic agents, combined with targeted sequencing and network analysis. Their results revealed a positive correlation between ex vivo drug responses and patients’ clinical outcomes, with a particularly strong association with overall survival compared to progression-free survival. These findings present the potential of combining functional precision medicine approaches with comprehensive molecular profiling to enhance treatment selection and improve the clinical management of lung cancer.

Another contribution to this Special Issue is a review conducted by Rodgers et al. [5], which discusses the impact of standard-of-care (SOC) treatment on glioblastoma, the most common malignant brain tumor in adults. Despite the use of SOC treatment, including surgery, radiation therapy (RT), and chemotherapy, survival rates remain alarmingly low, underscoring the urgent need for more effective therapeutic strategies. The authors also highlight the disappointing outcomes of numerous clinical trials over the past two decades, raising critical concerns about the translational relevance of current preclinical models. These models typically rely on treatment-naïve tumors, which do not reflect the clinical reality in which patients receive SOC therapy prior to recurrence. Since recurrent glioblastomas often exhibit distinct molecular changes driven by treatment-induced selection pressures, the authors advocate for the incorporation of SOC treatments into preclinical models to better simulate the recurrent disease state. This approach could significantly enhance the predictive value of preclinical studies and improve the success rate of future therapeutic interventions. To support this translational alignment, the authors developed and tested a fluorescence-guided resection technique in mouse models of glioblastoma [6]. Their findings suggest that this method is a valuable tool for testing new therapies and better understanding tumor behavior after surgery.

An immunotherapeutic strategy is proposed by Yang et al. [7], which explores the efficacy of an in vitro-generated CD103* dendritic cell vaccine (cDCV) derived from K7M3 osteosarcoma (OS) cell lysates. The authors demonstrate that cDCV, when administered intratumorally in a bilateral tumor model, not only inhibited the treated primary tumor but also suppressed the growth of untreated contralateral tumors, indicating the induction of a systemic immune response. This therapeutic effect was associated with a marked increase in T-cell infiltration in tumors and tumor-draining lymph nodes. Furthermore, systemic administration of cDCV following surgical removal of the primary tumor significantly reduced the size and number of established lung metastases. When combined with immune checkpoint blockades via anti-CTLA-4, the vaccine’s efficacy was further enhanced. These findings underscore the potential of cDCV as a novel immunotherapy for relapsed or metastatic OS, exemplifying how personalized immunomodulatory approaches can address the challenges of systemic disease progression in cancer.

Springer et al. [8] provide a comprehensive review of the mechanisms of cuproptosis and its potential implications for human health and disease, including cancer therapy. Copper is an essential trace element involved in numerous cellular processes, particularly as a cofactor for enzymes that catalyze vital biochemical reactions. However, maintaining copper homeostasis is critical, as both a deficiency and excess of copper can lead to cytotoxic effects. When intracellular copper concentrations exceed physiological thresholds, cells may undergo cuproptosis, also known as copper-induced cell death or copper toxicity. Copper ionophores, such as elesclomol and disulfiram, facilitate copper uptake into cells by forming complexes that transport copper across the cell membrane, specifically to mitochondria. This influx of copper increases intracellular levels, leading to oxidative stress, the disruption of mitochondrial respiration, and the inhibition of protein lipoylation, ultimately triggering cell death. These mechanisms highlight the potential of copper ionophores as therapeutic agents in cancer treatment. This review identifies potential protein targets and biomarkers, such as chaperone proteins, membrane-associated proteins, and intracellular enzymes, that may mediate copper toxicity and serve as candidates for therapeutic exploitation. The role of copper is also examined in the context of various cancers, including non-small cell lung cancer (NSCLC), colorectal cancer, prostate cancer, and uveal melanoma, to support the development of targeted therapies using copper-based nanomaterials, ionophores, or chelators.

With increasing global longevity, there has been a notable rise in the consumption of dietary supplements, particularly among adults, with the purpose of promoting overall well-being. Two contributions in this Special Issue address the implications of dietary supplementation in oncology. Jabbari et al. [9] present studies exploring the associations between dietary supplements and cancer incidence and mortality. Their analysis highlights specific supplements linked to increased cancer risk: vitamins A and E, calcium, selenium, zinc, and omega-3 for prostate cancer; vitamins A, B6, and B12 for lung cancer; and vitamin B9 for colon cancer. The authors emphasize the urgent need for more population-based studies and long-term clinical trials to assess the potential adverse effects of prolonged supplement use, including carcinogenic risks. On the other hand, Muranaka et al. [10] summarize recent evidence on how glutamine supplementation may enhance anti-cancer immune responses and the epigenetic regulation of tumor cells.

In summary, this Special Issue highlights the multifaceted advances in personalized medicine for cancer research, emphasizing the critical role of preclinical studies in bridging the gap between molecular insights and clinical application. These studies collectively reinforce the notion that personalized approaches are not only reshaping our understanding of cancer biology, but also offering tangible avenues to improve patient outcomes and quality of life.
